# Hematoma Intramural da Artéria Pulmonar Associado a Dissecção Aórtica Aguda

**DOI:** 10.36660/abc.20200775

**Published:** 2021-06-08

**Authors:** Lucas de Pádua Gomes de Farias, Ana Cristina Favaretto, Luciana de Pádua Silva Baptista, Gustavo Borges da Silva Teles

**Affiliations:** 1 UnitedHealth Group Brazil São PauloSP Brasil UnitedHealth Group Brazil , São Paulo , SP - Brasil

**Keywords:** Dor no Peito, Hematoma, Artéria Pulmonar, Angiografia por Tomografia Computadorizada/métodos, Aneurisma Dissecante

**Figura 1:**
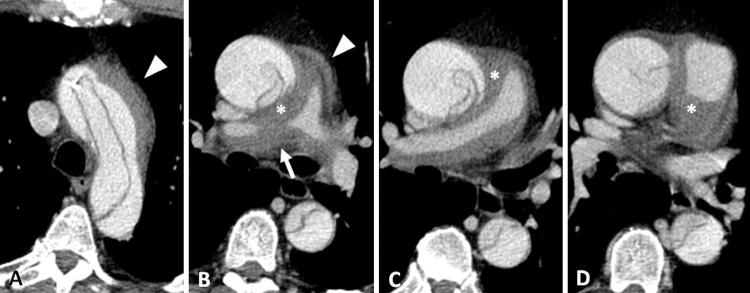
– Angiotomografia computadorizada de tórax, em aparelho com 16 fileiras de detectores (A a D – reconstrução multiplanar axial), evidencia dissecção aórtica aguda (tipo A de Stanford) associada a hematoma secundário (asterisco), envolvendo o tronco da artéria pulmonar e seus ramos principais, mais evidente à direita, determinando redução luminal da sua porção proximal (seta branca). Note também o hematoma mediastinal nas regiões para-aórtica e subaórtica (cabeça de seta branca).

Paciente do sexo masculino, 54 anos, tabagista, é admitido na unidade de emergência com dor torácica aguda e dispneia. Procedeu-se a investigação por meio da angiotomografia computadorizada de tórax que evidenciou extensa dissecção da aorta torácica com início no segmento ascendente (tipo A de Stanford) associada a hematoma intramural do tronco da artéria pulmonar e dos seus ramos principais, mais evidente à direita, que determina redução luminal pulmonar local, além de um pequeno hematoma mediastinal para-aórtico e subaórtico. Não havia sinais de tromboembolismo pulmonar e a avaliação do parênquima não evidenciou sinais de hemorragia pulmonar ( Figuras 1 e 2 ).

**Figura 2:**
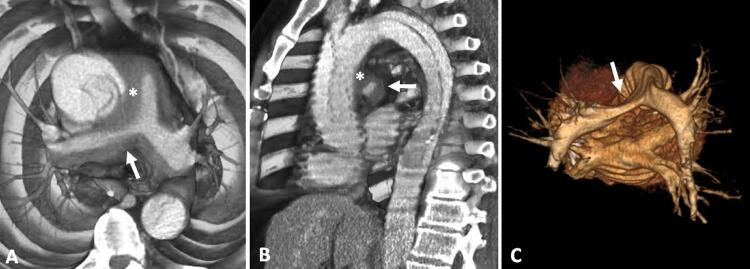
– Reconstruções tridimensionais de angiotomografia computadorizada de tórax, em aparelho com 16 fileiras de detectores, evidenciam dissecção aórtica aguda (tipo A de Stanford) associada a hematoma secundário (asterisco) envolvendo o tronco da artéria pulmonar e seus ramos principais, mais evidente à direita, determinando redução luminal da sua porção proximal (seta branca).

**Figura 3:**
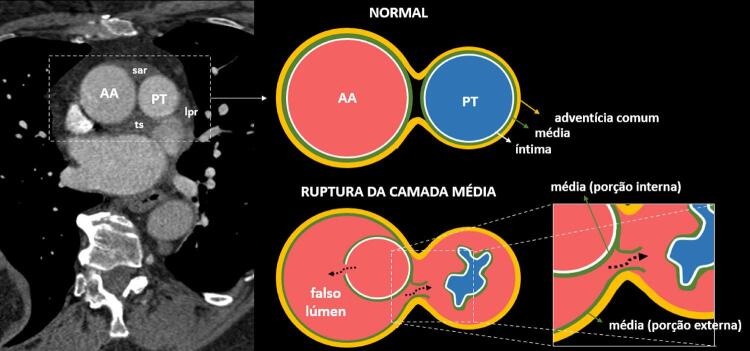
– Desenho esquemático evidencia a ruptura da porção mais externa da camada média no falso lúmen da dissecção aórtica, adjacente à artéria pulmonar, resultando extravasamento de sangue na adventícia comum entre a aorta e a artéria pulmonar que pode estreitar o lúmen arterial pulmonar. AA: aorta ascendente; lpr: recesso pulmonar esquerdo do seio transverso; PT: tronco pulmonar; sar: recesso aórtico superior; ts: seio transverso. Adaptado de Roberts. 5

A dissecção aórtica aguda é uma condição de alto risco de vida e o hematoma mediastinal que disseca a bainha das artérias pulmonares é considerado uma rara complicação ^1 - 3^ que pode simular tromboembolismo pulmonar e vasculites. ^4^ Isso geralmente ocorre porque, ao nível logo acima da válvula aórtica, a aorta ascendente e o tronco da artéria pulmonar compartilham uma adventícia comum, que se torna o pericárdio visceral caudalmente. ^1 , 4 , 5^ Na maioria dos casos, há a ruptura da cama média adjacente à artéria pulmonar direita, e o sangue flui da aorta ascendente para o espaço intersticial que limita as artérias pulmonares (hematoma intramural) ( Figura 3 ), podendo se estender aos septos interlobulares ou mesmo aos alvéolos por meio do interstício peribroncovascular. ^1 , 2 , 4^ Alguns casos isolados de hematoma da artéria pulmonar podem estar relacionados à patência do ducto arterioso, hipertensão pulmonar e desordens do tecido conjuntivo. ^6 - 9^
