# Supplementary material for: Hematoma Intramural da Artéria Pulmonar Associado a Dissecção Aórtica Aguda
Source: Arq Bras Cardiol. 2021 Jun 8;116(6):1169–71. [Article in Portuguese] doi: 10.36660/abc.20200775 (PMC8288521; doi:10.36660/abc.20200775)
Supplement: Translation

# Translation: Pulmonary Arterial Intramural Hematoma Due to Acute Aortic Dissection

**Authors:** Lucas de Pádua Gomes de Farias, Ana Cristina Favaretto, Luciana de Pádua Silva Baptista, Gustavo Borges da Silva Teles

**Affiliations:** 1 Brazil UnitedHealth Group Brazil, São Paulo, SP - Brazil

**Keywords:** Chest Pain, Hematoma, Pulmonary Artery, Computed Tomography Angiography/methods, Aneurysm, Dissecting

A 54-year-old male patient, who was a smoker, was admitted to the emergency room with acute chest pain and dyspnea. Investigation was initiated by means of computed tomography angiography of the chest, which showed extensive dissection of the thoracic aorta, beginning in the ascending segment (Stanford type A), associated with intramural hematoma of the pulmonary artery trunk and its main branches. It was more evident on the right, which determines local pulmonary luminal reduction, in addition to a small para-aortic and subaortic mediastinal hematoma. There were no signs of pulmonary thromboembolism, and the evaluation of the parenchyma showed no signs of pulmonary hemorrhage ( Figures 1 and 2 ).

**Figure 1:** – 16-row multi-detector computed tomography angiography of the chest (A to D – axial multiplanar reconstruction), shows acute aortic dissection (Stanford type A) associated with secondary hematoma (asterisk), involving the pulmonary artery trunk and its main branches. It is more evident on the right, determining luminal reduction of its proximal portion (white arrow). Note, as well, the mediastinal hematoma in the para-aortic and subaortic regions (white arrowhead).

**Figure 2:** – Three-dimensional reconstructions of a 16-row multi-detector computed tomography angiography, show acute aortic dissection (Stanford type A) associated with secondary hematoma (asterisk), involving the pulmonary artery trunk and its main branches. It is more evident on the right, determining luminal reduction of its proximal portion (white arrow).

Acute aortic dissection is a life-threatening condition, and mediastinal hematoma dissecting the pulmonary artery sheath is considered a rare complication,^1 - 3^ which can simulate pulmonary thromboembolism and vasculitis.^4^ This generally occurs because, at the level just above the aortic valve, the ascending aorta and the pulmonary artery trunk share a common adventitia, which caudally becomes the visceral pericardium.^1 , 4 , 5^ In most cases, there is a rupture of the middle bed adjacent to the right pulmonary artery, and blood flows from the ascending aorta into the interstitial space that limits the pulmonary arteries (intramural hematoma) ( Figure 3 ), and this can extend into the interlobular septa, or even the alveoli, through the peribronchovascular interstitium.^1 , 2 , 4^ Some isolated cases of pulmonary artery hematoma may be related to patent ductus arteriosus, pulmonary hypertension, or connective tissue disorders.^6 - 9^

**Figure 3:** – Schematic illustration showing the rupture of the outermost portion of the media layer in the false lumen of the aortic dissection, adjacent to the pulmonary artery, resulting in leakage of blood into the common adventitia between the aorta and the pulmonary artery, which can narrow the pulmonary arterial lumen. AA: ascending aorta; lpr: left pulmonary recess of the transverse sinus; PT: pulmonary trunk; sar: superior aortic recess; ts: transverse sinus. Adapted from Roberts. 5
